# An Opposite Effect of the CDK Inhibitor, p18^INK4c^ on Embryonic Stem Cells Compared with Tumor and Adult Stem Cells

**DOI:** 10.1371/journal.pone.0045212

**Published:** 2012-09-26

**Authors:** Yanxin Li, Rekha Pal, Li-Ying Sung, Haizhong Feng, Weimin Miao, Shi-Yuan Cheng, Cindy Tian, Tao Cheng

**Affiliations:** 1 State Key Laboratory of Experimental Hematology, Institute of Hematology and Blood Diseases Hospital, Center for Stem Cell Medicine, Chinese Academy of Medical Sciences and Peking Union Medical College, Tianjin, China; 2 Department of Radiation Oncology, University of Pittsburgh School of Medicine, Pittsburgh, Pennsylvania, United States of America; 3 Department of Pathology, University of Pittsburgh School of Medicine, Pittsburgh, Pennsylvania, United States of America; 4 University of Pittsburgh Cancer Institute, Pittsburgh, Pennsylvania, United States of America; 5 Institute of Biotechnology, National Taiwan University, Taipei, Taiwan; 6 Center for Regenerative Biology, Department of Animal Science, University of Connecticut, Storrs, Connecticut, United States of America; Kanazawa University, Japan

## Abstract

Self-renewal is a feature common to both adult and embryonic stem (ES) cells, as well as tumor stem cells (TSCs). The cyclin-dependent kinase inhibitor, p18^INK4c^, is a known tumor suppressor that can inhibit self-renewal of tumor cells or adult stem cells. Here, we demonstrate an opposite effect of p18 on ES cells in comparison with teratoma cells. Our results unexpectedly showed that overexpression of p18 accelerated the growth of mouse ES cells and embryonic bodies (EB); on the contrary, inhibited the growth of late stage teratoma. Up-regulation of ES cell markers (i.e., Oct4, Nanog, Sox2, and Rex1) were detected in both ES and EB cells, while concomitant down-regulation of various differentiation markers was observed in EB cells. These results demonstrate that p18 has an opposite effect on ES cells as compared with tumor cells and adult stem cells. Mechanistically, expression of CDK4 was significantly increased with overexpression of p18 in ES cells, likely leading to a release of CDK2 from the inhibition by p21 and p27. As a result, self-renewal of ES cells was enhanced. Our current study suggests that targeting p18 in different cell types may yield different outcomes, thereby having implications for therapeutic manipulations of cell cycle machinery in stem cells.

## Introduction

Embryonic stem (ES) cells are pluripotent cells with the capacity to self-renew and differentiate into different tissues/cell types present in three germ layers [1], [2]. Tumor cells, especially tumor stem cells (TSCs) or tumor-initiating cells (TICs) are also hypothesized to exhibit similar self-renewal characteristics [3], [4]. Moreover, a subset of TSCs have been reported to express high levels of ES cell marker genes, including Oct4 and Nanog [5], [6], [7], which have been associated with cancer resistance and relapse [5], [8]. Although similarities between ES cells and TSCs may provide a new opportunity to further understand the tumorigenic process, the tumorigenic potential of ES cells also represents a significant hurdle for their therapeutic applications. Thus, defining molecular targets that allow stemness to be dissociated from tumorigenesis is an important goal in ES cell biology, as well as tumor cell biology.

Stem cells constantly face the choices of self-renewal, differentiation, migration, quiescence and cell death [9]. Cell cycle regulation is one of the fundamental processes modulating cell fate choices and it represents a unique angle to dissect the relationship between tumorigenesis and stemness [10], [11], [12]. Cell cycle is primarily driven by cyclin-dependent kinases (CDKs), and CDKs are largely inhibited by CDK inhibitors (CKIs) including the INK4 family and the Cip/Kip family (seven members in total) in mammalian cells [13]. During the G1 phase, CDK4 or 6 and CDK2 act sequentially to drive the cell toward S phase. The INK4 family, including p15^Ink4b^ (p15), p16^Ink4a^ (p16), p18^Ink4c^ (p18), and p19^Ink4d^ (p19), specifically suppresses CDK4 or CDK6. In contrast, the Cip/Kip family, including p21^Cip1^ (p21), p27^Kip1^ (p27), and p57^Kip2^ (p57) broadly interacts with multiple types of CDK. However, p21 and p27 were also shown to promote the assembly of active kinase CDK4 or CDK6 complexes whereas they inhibits CDK2 activity [14]. Many types of adult stem cells, such as hematopoietic stem cells (HSCs), undergo a long quiescent stage Go phase that is mediated by distinct regulatory mechanisms involving p21 [15], [16], [17] or p57 [18] in a context-dependent manner. In contrast, ES cells typically exhibit a short G1 phase (approximately 1.5 h in mouse ES cells), primarily owing to high CDK2 activity that mediates self-renewing proliferation whereas pluripotent differentiation potential is maintained [19]. Moreover, previous studies have indicated that irreversible disruption of INK4 proteins, such as p16 or p15, coupled with p53 and RB pathways, may contribute to the formation of TSCs, thereby leading to tumorigenesis [10], [11].

p18, an INK4 family member, suppresses CDK4 or CDK6 during the G1 stage in somatic cells. It is a known haploinsufficient tumor suppressor and inhibits the self-renewal of adult stem cells [11]. p18 is detectable as early as the E7 embryo and widely expressed during later mouse embryogenesis [20]. p18 is also broadly present in many adult tissue types, including hematopoietic cells [21]. In contrast, there is virtually little expression of p18 and almost no detectable CDK4-associated activity of p18 protein in mouse ES cells [22]. Correspondingly, loss of p18 results in widespread hyperplasia and organomegaly after birth of the mice. The animals deficient in p18 develop both spontaneous and carcinogen-induced tumors in multiple organs [23], [24], [25], [26]. Moreover, as shown in mice [27], the correlation of p18 mutation with human glioblastoma further establishes p18 as a tumor suppressor in human [28]. We previously demonstrated that absence of p18 enhances the renewal of HSCs, leading to an increased number of HSCs [16], [29]. However, p18 null T cell leukemia was shown to be transformed in the T cell compartment, not at the level of HSCs [30]. A role of p18 in lung and breast cancer stem cells was also reported [31], [32].

In our current study, genetic manipulations of p18 were performed in a series of embryonic models to define the effect of p18 in ES cell growth as opposed to the previous documented roles of p18 in adult stem cells and tumor cells.

## Results

### “Gain-of-function” and “loss-of-function” Models of p18 in Mouse ES Cell Lines

**Figure 1:**
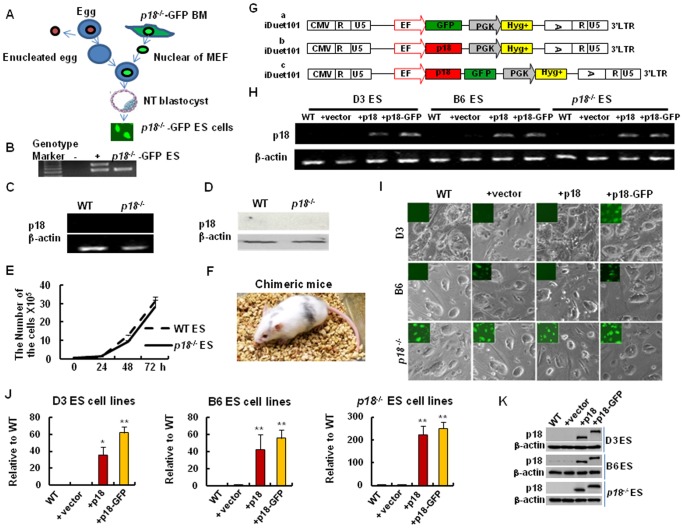
Establishment of “loss-of-function” and “Gain-of-function” models. (A) Generation of *p18^−/−^* ES cells by nuclear transfer. Briefly, the nuclei of *p18^−/−^* BM cells were microinjected into enucleated oocytes, and nuclear transfer (NT) embryos developed into blastocysts. These blastocyts were selected for derivation of *p18^−/−^* ES cells. (B) The genotype analysis of *p18^−/−^* ES cell line. (C) RT-PCR assays to detect mRNA levels of p18 in wild type (WT) and *p18^−/−^* ES cells. (D) Protein expression of p18 in WT and *p18^−/−^* ES cells detected by western blotting. β-actin was used as a loading control. (E) Growth curves for WT and *p18^−/−^* ES cells determined by counting the number of cells present at each time point using trypan blue staining. (F) Chimeric mice were generated by injecting *p18^−/−^* ES cells into diploid blastocysts. Reconstituted embryos were then developed in the uteri of foster mothers and chimera pups were obtained 19 days after injection. (G) Schematic representation of the lentiviral vectors used in this study. The vector, iDuet101, contains an EF1 promoter that drives the expression of GFP, p18, or a p18-GFP fusion protein. CMV, cytomegalovirus; R, repeat region in the viral long terminal repeat; U5 regions in the viral long terminal repeat; EF, elongation factor 1α; GFP, green fluorescent protein gene; PGK, mouse phosphoglycerate kinase promoter; *Hyg+*, hygromycin resistance gene; LTR, long terminal repeat of lentiviral DNA. (H) RT-PCR detection of mRNA levels in mouse ES cells transduced with p18-GFP or p18. Briefly, transduced cells for both groups were selected with hygromycin-B (I), and then infected with the iDuet101-GFP, as well as iDuet101-p18, or p18-GFP, lentiviruses. Top panel (D3 ES), middle panel (B6 ES), and lower panels (*p18^−/−^* ES) represent bright field images obtained, as well as fluorescence microscopy images added as inserts. WT: parental ES cells; +vector: iDuet 101-GFP; +p18: iDuet 101-p18; and +p18 - GFP: iDuet 101-p18 - GFP transduced ES cells. (J) Real-time RT-PCR detection of p18 mRNA in mouse ES cells transduced with or without p18 or p18-GFP. Data were analyzed according to the ΔC_T_ method. Values are expressed as the mean ± SD from two independent experiments, and all values were normalized to levels of β-actin. (K) Western blot analysis for p18 expression in three different ES cell lines with or without p18 or p18-GFP overexpression. *, p18-GFP. In B-E, H-K, data represent three independent experiments with similar results.

To determine the role of p18 in ES cell growth versus tumor growth, *p18^−/−^* ES cells labeled with green fluorescent protein (GFP) were derived from *p18^−/−^* GFP transgenic mice (Fig. 1A- B). In both *p18^−/−^* ES cells and *p18^+/+^* ES cells, levels of p18 mRNA and protein were undetectable (Fig. 1, C-D). Furthermore, no difference in cell growth was observed for *p18^−/−^* ES cells versus WT ES cells (Fig. 1E). *p18^−/−^* ES cells were then injected into blastocysts that were transplanted into ICR pseudo-pregnant recipient females, and chimera mice were successfully generated (Fig. 1F). Based on these data, it appears that deletion of p18 has no overt effect on mouse ES cells, thus reinforcing the notion that p18 is not required for the maintenance of mouse ES cells.

To investigate whether overexpression of p18 inhibits ES cell growth, p18 and a p18-GFP fusion protein were each overexpressed in wild type backgrounds (e.g., B6 ES cell lines came from C57BL/6 and D3 ES cell line was derived from 129S2/SvPas background) or *p18^−/−^* ES cells (C57BL/6 background). For these studies, ES cells were transduced with a lentiviral-GFP vector as a control, and compared with ES cells transduced with lentiviral-p18, or lentiviral-p18-GFP, vectors (Fig. 1G). Forty-eight hours post-infection, stably infected cells were selected with hygromycin B (110 µg/ml) for 7 d (Fig. 1I). Subsequent RT-PCR assays detected a 40-fold increase in p18 mRNA levels in D3 and B6 ES cells transduced with p18, and a >250-fold increase in p18 mRNA in *p18^−/−^* ES cells transduced with p18, relative to controls (Fig. 1H &1J). p18 overexpression in p18-GFP or p18 alone also were confirmed by the Western blot assay (Fig. 1K).

### Inhibition of Teratoma Growth by Ectopic Expression of p18

**Figure 2:**
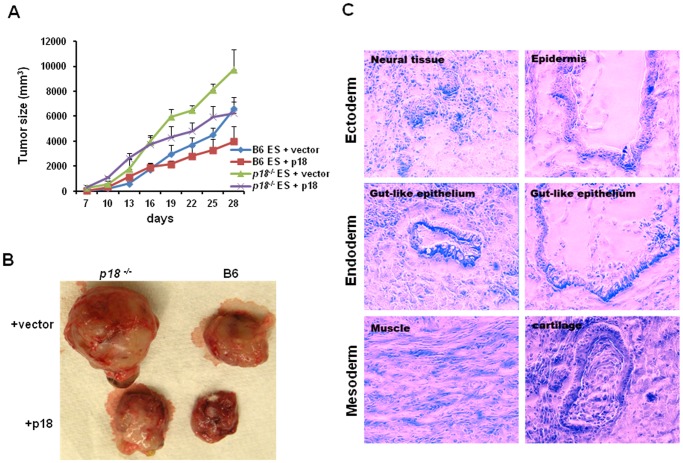
Ectopic expression of p18 inhibits teratoma formation of mouse ES cells. (A) Growth curve of teratoma obtained. Tumor size was monitored daily, then documented once tumor growth became visible (e.g., days 7–28). The graph represents the tumor growth volume observed for the different groups (as labeled) at different time points until the tumors were excised. (B) Approximately one month after implantation, tumors were excised and weighed. Representative images of one set of tumors derived from p18 mouse ES and control vector mouse ES are shown. (C) H&E staining of teratoma sections. All three germ layers were detected (e.g., ectoderm, endoderm and mesoderm). In A-C, data represent three independent experiments with similar results.

p18 has been shown to be a tumor suppressor in a variety of tumors [26]. To determine the role of p18 in teratoma growth, p18 and GFP transgene in B6 and *p18^−/−^* ES cell lines were injected into 5-week-old NOD/SCID mice. The injected mice were observed daily for teratoma formation and tumors were excised one month after injection. In these studies, tumor volume from p18-overexpressing ES cells was found to be significantly reduced compared to the control group (Fig. 2, A & B). Tumor sections of each p18-overexpressing teratoma generated were also analyzed using H&E staining. For each teratoma, the endoderm, ectoderm, and mesoderm germ lines were detected (Fig. 2C). In combination, these results suggest that p18 inhibits teratoma growth, which is consistent with a role for p18 as a tumor suppressor in somatic tissues [23].

### Enhancement of ES Cell Growth by Ectopic Expression of p18

**Figure 3:**
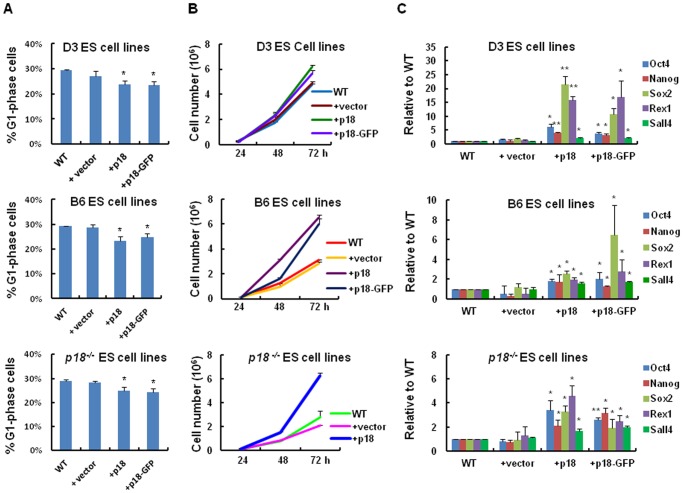
p18 stimulates ES growth associated with upregulation of stemness genes. (A) The percentage of G1-phase cells in D3, B6 and *p18^−/−^* cell lines with or without p18 overexpressing. (B) Growth curves plotted for WT, vector control, p18-overexpressing, and p18-GFP-overexpressing ES cells at different time points. (C) Real-time PCR assays of *Oct4*, *Nanog*, *Sox2*, *Rex1*, and *Sall4* were also performed for all three ES cell lines (e.g., D3, B6 and *p18^−/−^*). Data were analyzed according to the ΔC_T_ method and values were normalized to β-actin. Values are expressed as the mean ± SD. In A-C, data represent three independent experiments with similar results.

To investigate whether ectopic expression of p18 inhibits ES cell stemness, cell cycle and growth curve rates were determined for all the groups (overexpression of p18, p18-GFP and GFP in B6, D3 and *p18^−/−^* mouse ES cell lines) at various time points (24, 48 and 72 hrs) (Fig. 3A and 3B). Based on these assays, a significant increase in the growth rate of ES cells overexpressing p18 relative to GFP control or non-transduced ES cells was observed. Specifically, the growth rates for D3 ES cells, B6 ES cells, and *p18^−/−^* ES cells overexpressing p18 were 1.3-fold, 2.1-fold, and 2.3-fold higher than that of controls cells (Fig. 3B). These results indicate that p18 is a positive regulator for ES cell proliferation.

Transcription factors that have been shown to maintain the pluripotency and self-renewal of ES cells and tumor cells include Oct4, Nanog, Sox2, Rex1 and Sall4. Therefore, mRNA levels of these transcription factors were measured in D3, B6, and *p18^−/−^* cell lines. As shown in Figure 3C, overexpression of p18 was associated with significant increases in the expression of this panel of ES genes.

**Figure 4:**
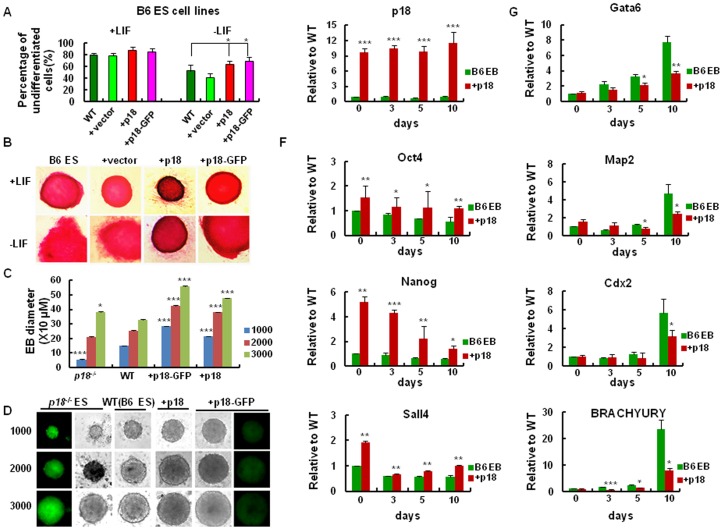
Early differentiation of ES cells is inhibited by ectopic expression of p18. (A, B) Comparison of the undifferentiated colonies presented in WT and p18-overexpression B6 ES cells analyzed by AP staining. Experiments were performed in triplicate in the presence or absence of leukemia inhibitory factor (LIF) in transduced and non-tranduced ES cells (B6 background). (A) The left panel (+LIF) and right panel (-LIF) represent bright field images of differentiated ES cells generated by cultivating ES cells in the presence or absence of LIF for 5 d. (B) Representative images of the differentiation morphology associated with transduced, and non-transduced, ES cells (B6 background). (C) Comparison of embroid body formation at day 5 after equal numbers of ES cells were plated at day 0 for all of the groups indicated. A mean diameter for these EB was determined from 4 measurements. Data represent the mean ± SD. (D) Representative bright field images, as well as fluorescence images, of EB grown in the presence of LIF for 5 d (E, F and G). Total RNA was extracted from EB at day 0, 3, 5, and 10. Expression of *p18*, *Oct4*, *Nanog*, *Sall4*, *Gata6*, *Map2*, *Cdx2*, and *BRACHYURY* were analyzed using real-time RT-PCR. Data were analyzed according to the ΔC_T_ method. All values were normalized to β-actin and expressed relative to WT levels. Values are expressed as the mean ± SD. In A to G, data represent three independent experiments with similar results.

To further investigate whether expression of p18 promotes the stemness of ES cells via inhibition of ES cell differentiation, cell morphology was examined by AP staining following induction of differentiation. In these assays, a decreased percentage of ES colonies overexpressing p18 were observed to undergo differentiation relative to WT and control ES cells (Fig. 4, A & B; and S1). In addition, a defined number of ES cells were seeded and differentiation of EB was assessed for its colony size and molecular markers (Fig. 4C-G). In these assays, the size of the EB observed in WT cells were smaller than that observed in ES cells overexpressing p18 (Fig. 4, C & D). Based on the increase in p18 expression observed in ES cells, mRNA levels of *p18* were also assayed in EB. Consistent with the previous results, levels of *p18* mRNA were found to be significantly up-regulated in the EB cells overexpressing p18, or p18-GFP, relative to control cells (Fig. 4E; and S2). To further characterize the differentiation of ES cells with or without p18, expression of self-renewal marker genes (e.g., *Oct4*, *Nanog*, and *Sall4*), as well as differentiation marker genes (e.g., *Gata6*, *Map2*, *Cdx2*, and *BRACHYURY*), were detected in EB using real-time RT-PCR. In the cells overexpressing p18, mRNA levels of *Oct4*, *Nanog*, and *Sall4* were expressed 3.7-fold, 3.1-fold, and 1.7-fold higher respectively, at day 10 than that in control and WT cells (Fig. 4F; and S2). In contrast, mRNA levels of *Gata6*, *Map2*, *Cdx2*, and *BRCHYURY* were found to be expressed 0.47-fold, 0.66-fold, 0.55-fold, and 0.33-fold lower, respectively, at day 10 than that in WT control cells (Fig. 4G; and S2). These results suggest that the differentiation process of EB was retarded in the presence of p18 overexpression.

### Involvement of CDK4/CDK2 in the Maintenance of Stemness Associated with Ectopic Expression of p18

**Figure 5:**
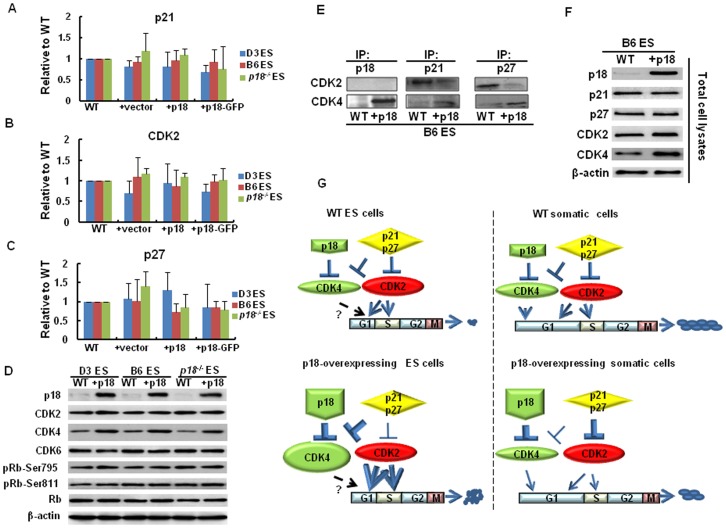
Reassortment of CDKI to CDKs by p18 overexpression in ES cells. (A, B and C) Real-time RT-PCR analysis of *p21*, *p27*, and *CDK2* mRNA levels. All values were normalized to β-actin. Values are expressed as the mean ± SD. (D) Western blotting performed to detect protein levels of p18, CDK2, CDK4, CDK6, pRb-Ser795, pRb-Ser811, and total Rb. Detection of β-actin was used as a loading control. (E) IP assays of p18, p21 and p27 were performed using cell lysate (100 µg total protein) from stably transduced p18, or WT ES cells. Immunocomplexes obtained were then immunoblotted with anti-CDK4 and anti-CDK2 antibodies. (F) Total protein extracts were obtained from stably transduced p18, or WT ES cells and immunoblotted with anti-p18, anti-p21, anti-p27, and anti-cdk2 and anti-cdk4 antibodies. β-actin was used as a loading control. (G) A model for a proposed mechanism by which p18 enhances the self-renewal of ES cells, while inhibiting their differentiation potential. Briefly, ectopic expression of p18 promotes overexpression of CDK4, which in turn enhances the association of p21 and p27 with CDK4, and ultimately upregulates CDK2 activities. As a result, the cell cycle is accelerated and the self-renewal is enhanced, whereas the differentiation process is slowed. In A to F, data represent three independent experiments with similar results.

The above results suggest that p18 can enhance the growth of mouse ES cells, yet inhibit tumorigenesis during teratoma formation. This disconnected function may derive from the different cell types involved. In somatic cells, the cyclin D-CDK4-6/INK4/Rb/E2F pathway plays a key role in controlling cell growth by integrating multiple mitogenic and anti-mitogenic stimuli [33]. Collectively, previous studies suggest that ectopic expression of p18 may inhibit tumorigenesis by binding to either CDK4, or CDK6, to inhibit the enzymatic activity and consequently block cell cycle progression. However, in ES cells, the function of the cyclin D-CDK4-6/INK4/Rb pathway has not been fully established whereas CDK2 is known to be a major driving force for cell cycle progression [19]. Thus, mRNA levels of *p21*, *p27*, and *CDK2* were assayed in the ES cells using real-time RT-PCR. In these assays, no change in the mRNA levels of *p21*, *p27*, or *CDK2* were observed relative to controls (Fig. 5, A–C). However, when protein levels of various cell cycle regulators were subsequently assayed, only levels of CDK4 were found to have significantly increased along with p18 overexpression (Fig. 5D). Immunoprecipitation (IP) assays were further performed to investigate whether interactions of p18, p21, and p27 with CDK2 or CDK4 were affected. When p18 was overexpressed in ES cells, binding of p18 to CDK4 was significantly increased relative to WT control cells. In contrast, p18 did not bind CDK2 in either ES cells overexpressing p18 or WT cells (Fig. 5, E & F). In addition, IP assays revealed a higher level of binding of p21 and p27 withCDK4, relative to CDK2, in the ES cells overexpressing p18 compared to WT control cells (Fig. 5, E & F).Taken together, these results suggest an paradigm in ES cells (Fig. 5G), where overexpression of p18 significantly up-regulates CDK4 expression (Fig. 5, D & F) and induces binding of p21 and p27 to CDK4 rather than CDK2. As a result, CDK2 activity is upregulated which in turn promotes cell cycle progression and enhances growth of ES cells.

## Discussion

While p18 has previously been characterized as a “negative regulator” of cell cycle progression and a suppressor of tumor growth, the results of our current study unexpectedly demonstrate that ectopic expression of p18 can enhance the growth of mouse ES cells concomitant with up-regulation of various embryonic markers (e.g., Oct4, Nanog, Sox2, and Rex1) and down-regulation of various differentiation markers (e.g., Gata6, Map2, Cdx2, and BRACHYURY). Further analysis also revealed that ES cell proliferation was accelerated via up-regulation of CDK4 when p18 was overexpressed. These results demonstrate that p18 stimulates the growth of ES cells, which is opposite to the previously documented roles of p18 in tumor or adult stem cells.

Notably, overexpression of p18 was also found to enhance the growth of EB (Fig. 4) whereas it inhibited the growth of teratoma (Fig. 2). However, despite the similarities that exist between teratoma and EB as ES progeny, a major distinction is their stage of differentiation. Since EB represent the early stage of ES differentiation and may contain undifferentiated ES cells, an enlarged size of EB should reflect an outcome of enhanced growth of ES cells by overexpression of p18. In contrast, the size of teratoma was measured 30 days after the inoculation of ES cells. At that time point, the teratoma formed would be in the late stage of ES cell differentiation, and should mainly be composed of more differentiated somatic cells. Therefore, the differential effects of p18 on teratoma and EB can be explained by stage-specific effects of p18 during ES differentiation. Like the effect of p18 on ES proliferation, a recent report also demonstrated a positive role of p18 in the proliferation of hematopoietic progenitor cells (measured by the colony-forming assay) [34]. In contrast, data from our previous studies, demonstrated that self-renewal of HSC is inhibited by p18 [16], [29], [30]. Taken together, p18 may function in cell type-specific and differentiation-specific manners.

Proper control of cell cycle progression is of critical importance for regulating all the cell types. Interestingly, cell cycle control in ES cells has been shown to be independent of the regulatory effects of the Rb and p53 pathways [35], [36]. For example, ectopic expression of p16, another prominent CDK4/6 cyclin D inhibitor, does not inhibit the proliferative capacity of mouse ES cells [37]. In addition, ES cells do not exhibit growth arrest in the G1 phase [37]. Based on the results of the present study where ectopic expression of p18 in mouse ES cells enhanced the cell growth and stemness by up-regulating self-renewal genes and down-regulating differentiation genes, it further reinforces the notion that cell cycle regulation in ES cells is distinct from that in somatic cells and cell cycle regulators have distinct effects on ES cells vs. somatic cells including adult stem cells and tumor cells.

Ectopic expression of p18 in mouse ES cells was associated with the up-regulation of CDK4 (Fig. 5, D & F), and enhanced binding of p18 to CDK4 (Fig. 5, E & F). However, because of the absence of evidence concerning how p18 directly induces up-regulation of CDK4, we hypothesize that a feedback mechanism exists among cell cycle regulators. In fact, several studies have demonstrated that feedback among CKIs can affect CDKs based on a reassortment of cyclin-CDK-CKIs complexes [28], [38]. Moreover, previous studies have confirmed that p18 directly interacts with CDK4 based on a comparison of p18 and CDK4 double knockout, and single knockout mouse models [39]. While CDK4 and CDK2 share a role in the G1/S transition of somatic cells, the role of CDK4 in ES cells has not been elucidated. Based on our current data and previous studies by others, a molecular paradigm concerning how p18 affects ES cells is proposed (Fig. 5G). Due to the resulted up-regulation of CDK4 by p18, p21 and p27 preferentially bind CDK4 rather than CDK2 (Fig. 5, E & F). As a result, the inhibition of CDK2 by p21 and p27 is reduced. Since CDK2, and not CDK4 or CDK6, is the major driving force for cell cycle progression in ES cells [40], decreased inhibition of CDK2 by p21 and p27 should accelerate cell proliferation. Thus, this model explains why overexpression of p18 enhances ES cell growth. According to a large body of previous studies, CDK4, together with CDK2, are a major driving force for cell cycle progression in somatic cells, the strong inhibition of CDK4 by p18 and intensive binding of p21 and p27 to CDK2 would result in significant inhibition of the cell growth (Fig. 5G) [38]. Therefore, targeting p18 in these different stem cell types may yield cell type-specific outcomes, thereby having therapeutic implications.

## Materials and Methods

### Cell Culture

ES-D3 (CRL-1934) and ES-C57BL-6 (B6, SCRC-1002) cell lines from ATCC (Manassas, VA) were cultured on a irradiated mouse embryonic fibroblast (MEF) cell line (CF-1Strain,Chemicon,Temecula, CA) in ES qualified Dulbecco’s Modified Eagle’s Medium (DMEM) supplemented with 15% fetal calf serum (FCS), 0.1 mM β-mercaptoethanol, glutamine, non-essential amino acids and 1000 U/ml recombinant human leukemia inhibitory factor (LIF) (ESGRO) (Chemicon, Temecula, CA) at 37°C under 5% CO_2_. For the culture of ES cells, the MEF were irradiated at 60 Gy and then plated on the gelatinized plates. Irradiated MEFs (2×10^5^ cells) were coated on the 6 well plates to support the culture of mouse ES cells. Primary MEFs (passage 3) were maintained in DMEM supplemented with 10% fetal bovine serum and 2 mM L-glutamine.

### Somatic Cell Nuclear Transfer (SCNT), Embryo Culture and Derivation of p18^−/−^ ES Cell Lines

SCNT was performed by direct nuclear injection as previously reported [41]. In brief, BDF1 (C57BL/6 X DBA/2) mice from Charles River Laboratories (Wilmington, MA) were used as the oocyte donors, and *p18^−/−^* GFP (C57BL/6) mice were used as nuclei donors. Nuclei of *p18^−/−^* GFP BM cells were directly injected into enucleated eggs. The reconstructed oocytes were cultured in CZB medium for 1–3 h and further placed into calcium-free CZB medium containing 10 mM strontium and 5 µg/ml cytochalasin B for 6 h of activation treatment. Activated oocytes were cultured in KSOM+ AA medium for 4 days at 37°C in a humidified atmosphere of 5% CO_2_, 5% O_2_, and 90% N_2_.

When cloned *p18^−/−^* GFP embryos reached the blastocyst stage after 4 days of culture, they were transferred into plates containing inactive primary mouse embryonic fibroblast (pMEF) feeder cells in ESC medium and cultured for about 7–10 days [42]. The ESC medium used for ESC derivation including Knockout-DMEM (Invitrogen, Carlsbad, CA) supplemented with 15% knockout serum replacement (KSR, Invitrogen), 1,000 U/ml leukemia inhibitory factor (LIF) (Invitrogen), 1% penicillin-streptomycin (Invitrogen), 1% L-glutamine (Specialty Media), 1% non-essential amino acids (Specialty Media), 1% nucleosides for ES cells (Specialty Media), 1% 2-mercaptoethanol (Specialty Media) and 6 mM PD98059 (Promega, Madison, WI). Newly formed inner cell mass outgrowths were mechanically dissociated using trypsin (Invitrogen, Carlsbad, CA) treatment and replated on pMEF feeder cells until stable cell lines established. This work was performed in Dr Jerry Yang’s laboratory at the University of Connecticut.

### Chimera Formation

The *p18^−/−^* ES cells were microinjected into ICR host blastocysts and transferred into 2.5-day post-coitum ICR pseudopregnant recipient females. Chimaerism was ascertained after birth by the appearance of black coat color (from p18^−/−^ ES cells) in white host pups.

### Lentiviral Vector Constructs

The iDuet101 lentiviral vector was kindly gifted by Dr. Linzhao Cheng (Johns Hopkins University). The vector constructs were made by inserting the full length p18 cDNA into iDuet101 by *Kpn* I digestion to get iDuet101-p18-GFP or by *Cla* I and *Kpn* I digestion to replace the GFP and get iDuet101-p18 (Fig 1G).

### Lentiviral Vector Production

Lipofectamine (Invitrogen, Carlsbad, California) transfection was used to generate the virus supernatant. In brief, 293T cells were transfected using the lipofectamine transfection reagent with the cytomegalovirus (CMV) R8.91 and pMD.G helper plasmids. The lipofectamine-DNA mixture was applied directly onto 293T cells. The medium was replaced at 20–24 hours post-transfection and the medium containing the viral particles was collected at 48 hours post-transfection, filtered through 0.45-µm filters for use and supplemented with 6-µg/ml polybrene and Leukemia inhibitory factor (LIF).

### Lentiviral Transduction on ES Cells

Mouse ES cells were grown on a feeder layer of irradiated MEFs in the presence of LIF. ES cells used for viral infection were washed, trypsinized and plated at a density of 10^6^ cells in the wells of a 6-well gelatin-coated dish, and viral supernatant was added for 4 hr to overnight in the presence of 5 µg/ml polybrene (Sigma, St Louis, MO) and LIF. Following viral infection, the ES cells were resuspended in fresh ES cell medium and grown on a new feeder layer of irradiated MEFs. At three days post-infection, hygromycin B was added to select for p18 overexpression ES cells and GFP positive cells.

### AP Staining

ES cells were cultured in medium with or without LIF and gently washed with 1X PBST (1X PBS with 0.05% Tween-20) before staining with stemTAG AP staining kit. Cells were fixed for 2 min at room temperature with fixing solution and washed twice with 1X PBST. After final washing, a freshly prepared stemTAG AP staining solution was added to the plate, and cells were incubated for 15 min at room temperature in the dark. The staining solution was removed, and the cells were washed with 1X PBS and photographed.

### Cell Cycle Analysis

Cell cycle analysis assays were performed using the Click-iT® EdU Assay Kit. We selected pacific blue to show the EDU and 7-AAD to stain DNA. The flow cytometry data were analyzed by the Syan software.

### Growth Curve

Cell growth curves were compared among the transduced p18 overexpressing ES cells, transduced GFP-expressing control ES cells and parental ES cells according to the method [43]. Briefly, 1.5×10^5^ ES cells were seeded in each well of a 12-well plate, and the growth curves were plotted by counting cells every 24 hours over a three day period with excel software.

### Formation of Embroid Bodies (EBs) in Suspension Culture

To induce ES cell differentiation into embroid bodies (EBs), undifferentiated ES cells (the parental, p18 overexpression and *p18^−/−^* ) were cultivated in DMEM supplemented with 15% FBS (specialty media, Chemicon), 1x non-essential amino acids, 2 mM Glutamine and 0.1 mM β-mercaptoethanol (specialty media, Chemicon) without LIF. Briefly, 1000, 2000, or 3000 ES cells were plated in differentiation medium depleted of LIF, poly (2-hydroxyethyl methacrylate)-coated 96-well plates that promoted the formation of floating cell aggregates. EBs were collected for RNA extraction at day 3, 5 and 10. EB cell morphology was compared among *p18^−/−^*, the parental and p18 overexpressing ES cells using photographs of the cells taken on day 5.

### Quantitative Real-time PCR Analysis

For the determination of mRNA levels of specific genes, undifferentiated ES cells were harvested by treating with trypsin-EDTA solution and washed with PBS three times. EBs were also harvested and washed 3 times with PBS. Total RNA was extracted by using RNeasy kit (Qiagen, Valencia, CA) according to the manufacturer’s instructions. RNA was treated with RNase–free DNase (Invitrogen, Carlsbad, California) for 15 min at room temperature before reverse transcription with superscript II RT (Invitrogen, Carlsbad, California). Real- time PCR was performed on the chromo 4™ detector (M J Research, Waltham, MA) with SYBR Green PCR master mix (DyNAmo ™ HS distributed by New England Biolabs Ipswich, MA). PCR conditions consisted of a 10-min hot start at 95°C followed by 40 cycles of 95°C for 15 sec, 60°C for 1 min and incubation for 3 sec at 77°C with a final extension for 10 min at 72°C. The average threshold cycle (Ct) for each gene was determined from triplicate reactions and the levels of gene expression relative to β-actin were determined as described [44]. Gene expression analysis for ES markers (Oct4, Sox2, Nanog, Rex1, Sall4) and differentiation markers (Cdx2, Brachyury, Gata6, Map2) were performed using published primers [43] (Table 1).

**Table 1:** Sequences of the Primers used for Real-time RT-PCR assays.

Gene	Sequence (5′ to 3′)	Reference
Oct4	Forward CTG AGG GCC AGG CAG GAG CAC GAGReverse CTG TAG GGA GGG CTT CGG GCA CTT	Takahashi *et al.,* 2006
Sox2	Forward GGT TAC CTC TTC CTC CCA CTC CAGReverse TCA CAT GTG CGA CAG GGG CAG	Takahashi *et al.,* 2006
Rex2	Forward ACG AGT GGC AGT TTC TTC TTG GGAReverse TAT GAC TCA CTT CCA GGG GGC ACT	Takahashi *et al.,* 2006
Nanog	Forward AGG GTC TGC TAC TGA GAT GCT CTGReverse CAA CCA CTG GTT TTT CTG CCA CCG	Takahashi *et al.,* 2006
p18	Forward TTA TGA AGC ACA CAG CCT GCA ATG TReverse ACG GAC AGC CAA CCA ACT AAC GG	Lu SJ *et al.,* 2002
p21	Forward TCA AAC GTG AGA GTG TCT AAC GGReverse CTC AGA CAC CAG AGT GC	Yang W *et al.,* 2003
p27	Forward GGG CAG ATA CGA GTG GCA GReverse CCT GAG ACC CAA TTA AAG GCA C	http: pga.mgh.harvard.edu/primerbank
CDK2	Forward TGTGCCTCCCCTGGATGAAGReverse CATCCTGGAAGAAAGGGTGA	
Gata6	Forward ACC TTA TGG CGT AGA AAT GCT GAG GGT GReverse CTG AAT ACT TGA GGT CAC TGT TCT CGG G	Takahashi *et al.,* 2006
Brachyury	Forward ATG CCA AAG AAA GAA ACG ACReverse AGA GGC TGT AGA ACA TGA TT	Takahashi *et al.,* 2006
Map2	Forward CAT CGC CAG CCT CGG AAC AAA CAGReverse TGC GCA AAT GGA ACT GGA GGC AAC	Takahashi *et al.,* 2006
Cdx2	Forward GGC GAA ACC TGT GCG AGT GGA TGC GGA AReverse GAT TGC TGT GCC GCC GCC GCT TCA GAC C	Takahashi *et al.,* 2006
Sall4	Forward AACATATGCGGGCGGGCCTTCAReverse CCAGGAGGCGGGGTCCACACTC	Takahashi *et al.,* 2006
β-actin	Forward GAA ATC GTG CGT GAC ATC AAA GReverse TGT AGT TTC ATG GAT GCC ACA G	

### Teratoma Formation

A total of 2×10^6^ ES cells or ES cell-derived differentiated cells resuspended in 100 µl PBS were injected subcutaneously into the dorsal flank of Avertin-anesthesized SCID mice. The injected mice were observed daily for any changes in their behavior or condition. Tumor sizes were measured every three days. At approximately four weeks post-injection, teratoma were surgically removed from the mice after CO_2_ euthanasia, measured, weighed, and snap-frozen, embedded in tissue-tek with O.C.T. compound, and stored at −80°C. The samples were sectioned at a thickness of 8 mm, and stained with haematoxylin and eosin (H&E) for pathological examination.

### Immunoblot Analysis

Immunoblot analysis was performed with the standard procedures. Briefly, mouse ES cells were washed with PBS, enzymatically dissociated with trypsin/EDTA (Gibco, Grand Island, NY) treatment, and finally collected in the modified lysis buffer (50 mM Tris–HCl, pH 7.4, 1% NP-40, 0.25% sodium deoxycholate, 150 mM NaCl, 1 mM EDTA, 2 mM sodium orthovanadate, 5 mM sodium fluoride, 1 mM PMSF, 1 mM DTT, 10 mg/ml leupeptin, and 2 mg/ml pepsatin A) allowing to be lysed for 60 min on ice. The resulting cell lysates were centrifuged for 15 min at 14,000×*g* at 4°C. Protein concentration of the supernatants was determined using the Bio-Rad protein assay. Samples containing equal amounts of proteins were subjected to 12% SDS–polyacrylamide gel electrophoresis and transferred to polyvinyl difluoride membranes (Bio-Rad, Hercules, CA). The blots were incubated with primary antibodies and then secondary antibodies followed by detection with the enhanced chemiluminescence (NEN Life Science Products, Boston, MA). Membranes were reprobed for the determination of two or more proteins. The antibodies against CDK4 (C-22), p21 (F-5), CDK2 (M2), CDK6 (H-230), p27 (C-19), Rb (M-153), and Polyclonal antibodies against pRb (Ser 807/811)-R) and pRb (Ser 795)-R) were from Santa Cruz Biotechnology, Inc., (Santa Cruz, CA). Anti-p18 (18P118) was purchased from NeoMarkers (Fremont, CA). A mouse antibody against ß-actin was from Santa Cruz Biotechnology, Inc., (Santa Cruz, CA).

### Immunoprecipitation

For immunoprecipitation (IP) experiments, 400 µg of cell lysates was adjusted to a volume of 400 µl with RIPA buffer supplemented with protease inhibitors and precleared with 30 µl of a 50% suspension of Protein A Sepharose (PAS) beads for 1 h at 4°C. The supernatant was then incubated overnight at 4°C in the presence of the primary antibody prior to tumbling with 30 ml of fresh PAS beads for an additional 2 h. The beads were washed three times in RIPA buffer, heated at 95°C in an equal volume of 2xSDS loading buffer (100 mM Tris-HCl pH 6.8, 20% glycerol, 4% SDS, 200 mM DTT and 0.2% bromophenol blue) and resolved on 12% Tris-tricine polyacrylamide gels for immunoblotting.

### Statistical Analyses

Data are expressed as mean ± SD. A *t* test was used to compare the mRNA expression levels of mouse p18 between the undifferentiated and differentiated ES cells. All analyses were 2-tailed and considered statistically significant when *P* values were less than or equal to 0.05.

### Study Approval

Studies using mouse materials about mouse ES and embryos were reviewed and approved by the Institutional Review Board of the University of Pittsburgh. No informed consent was required.

## Supporting Information

Figure S1 **p18 inhibits mouse ES cell differentiation.** Undifferentiated colonies were analyzed using alkaline phosphatase (AP) staining in the presence or absence of leukemia inhibitory factor (LIF) in transduced, as well as non-transduced, D3 ES cells and p18^−/−^ ES cells. Experiments were performed in triplicate.
(TIF)

Figure S2 **Ectopic expression of p18 maintains stem cell markers and inhibits differentiation of mouse EB cells.** Total RNA was extracted from D3 and p18^−/−^ EB at day 0, 3, 5, and 10, respectively. Using real-time PCR, mRNA levels of *p18*, *Oct4*, *Nanog*, *Sall4*, *Gata6*, *Map2*, *Cdx2*, and *BRACHYURY* were analyzed in undifferentiated ES cells relative to differentiated EB. Data were analyzed according to the ΔC_T_ method. All the values were normalized to β-actin and expressed relative to WT levels. Values are expressed as the mean ± SD.
(TIF)

Table S1 **Sequences of the Primers used for Real-time RT-PCR assays.**
(DOC)
