# Supplementary figures and images for: An Opposite Effect of the CDK Inhibitor, p18INK4c on Embryonic Stem Cells Compared with Tumor and Adult Stem Cells
Source: PLoS One. 2012 Sep 26;7(9):e45212. doi: 10.1371/journal.pone.0045212 (PMC3458833; doi:10.1371/journal.pone.0045212)

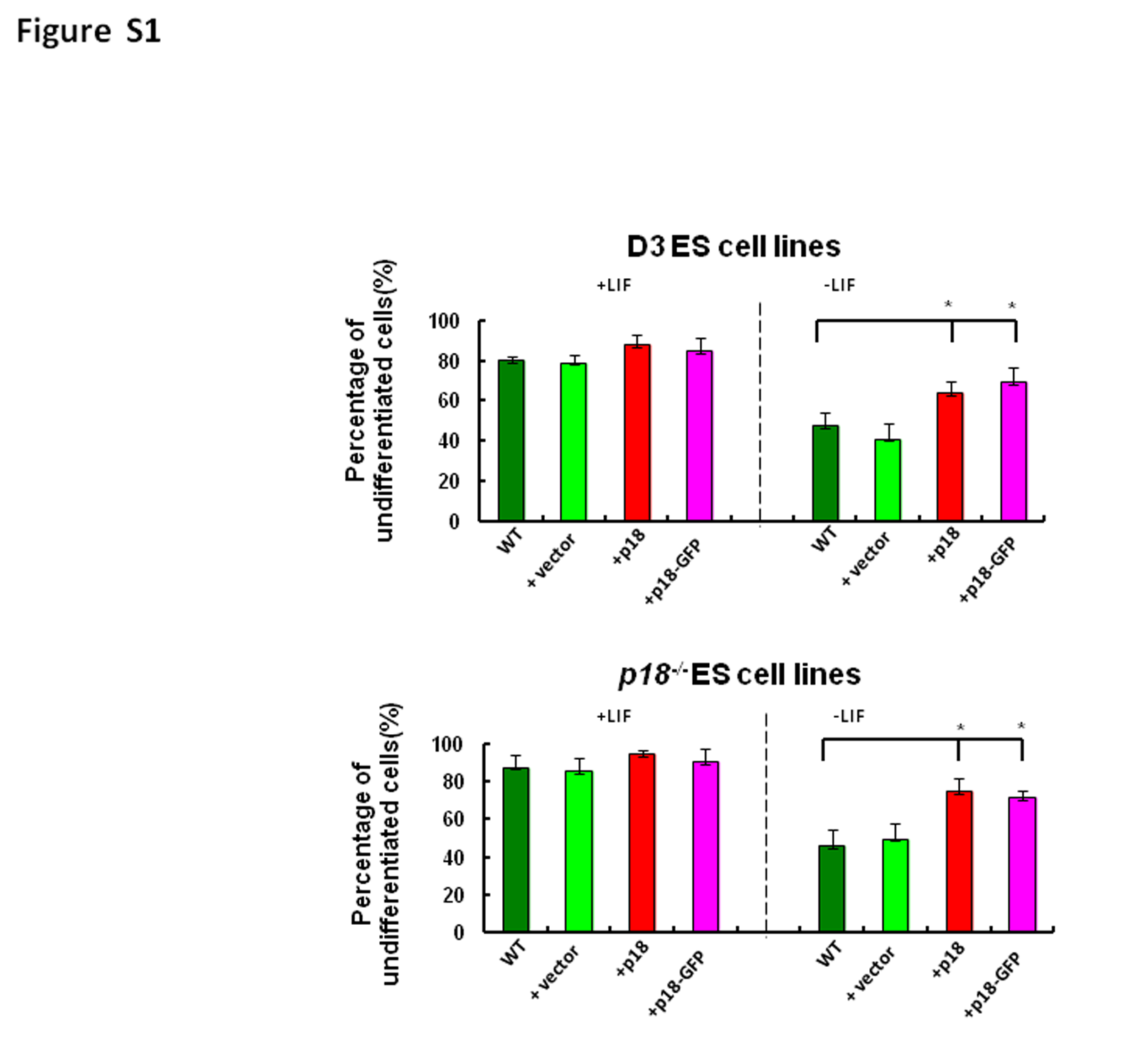

Supplement: Figure S1 — p18 inhibits mouse ES cell differentiation. Undifferentiated colonies were analyzed using alkaline phosphatase (AP) staining in the presence or absence of leukemia inhibitory factor (LIF) in transduced, as well as non-transduced, D3 ES cells and p18−/− ES cells. Experiments were performed in triplicate. (TIF) [file pone.0045212.s001.tif]

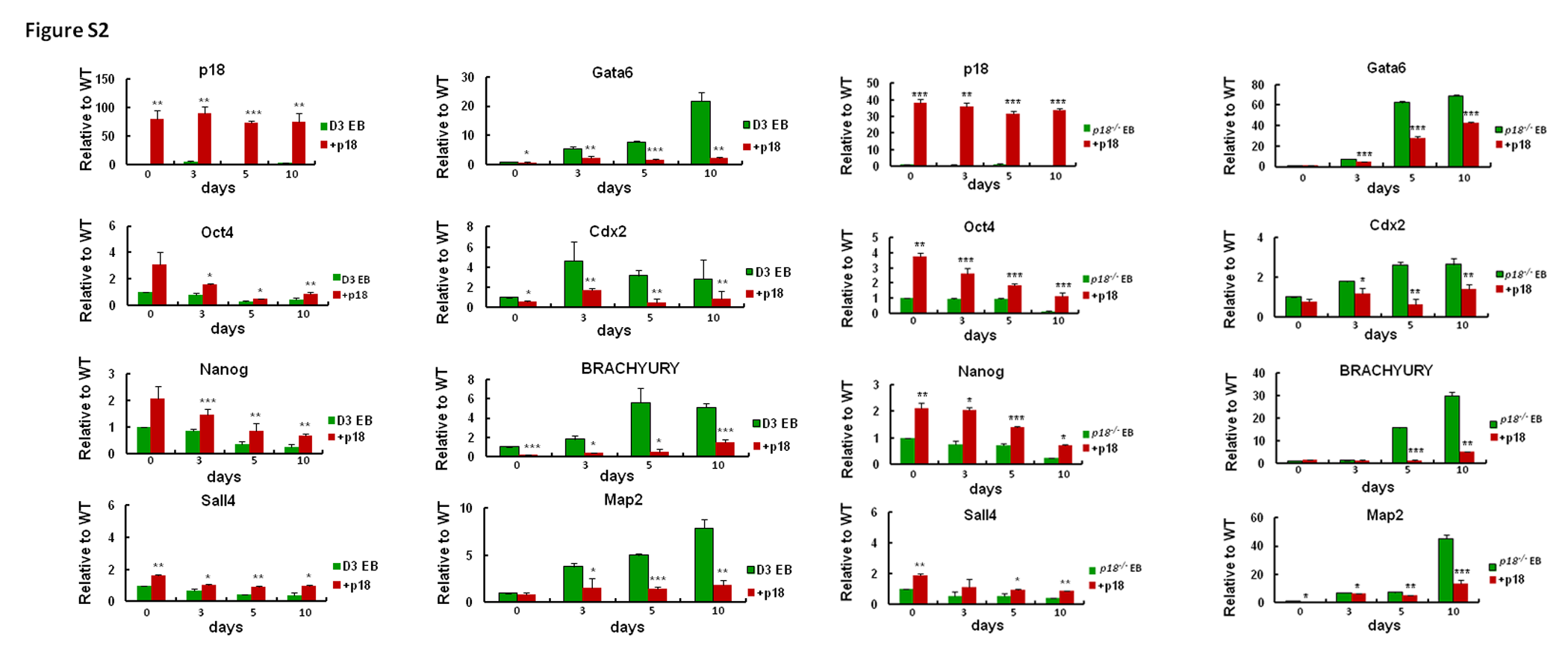

Supplement: Figure S2 — Ectopic expression of p18 maintains stem cell markers and inhibits differentiation of mouse EB cells. Total RNA was extracted from D3 and p18−/− EB at day 0, 3, 5, and 10, respectively. Using real-time PCR, mRNA levels of p18, Oct4, Nanog, Sall4, Gata6, Map2, Cdx2, and BRACHYURY were analyzed in undifferentiated ES cells relative to differentiated EB. Data were analyzed according to the ΔCT method. All the values were normalized to β-actin and expressed relative to WT levels. Values are expressed as the mean ± SD. (TIF) [file pone.0045212.s002.tif]
