# Supplementary material for: An Opposite Effect of the CDK Inhibitor, p18INK4c on Embryonic Stem Cells Compared with Tumor and Adult Stem Cells
Source: PLoS One. 2012 Sep 26;7(9):e45212. doi: 10.1371/journal.pone.0045212 (PMC3458833; doi:10.1371/journal.pone.0045212)
Supplement: Table S1 — Sequences of the Primers used for Real-time RT-PCR assays. (DOC) [file pone.0045212.s003.doc]

**Supplemental Table S1. Sequences of the Primers used for Real-time RT-PCR assays.**

| **Gene** | **Sequence (5' to 3')** | **Reference** |
| --- | --- | --- |
| Oct4  Sox2  Rex2  Nanog  p18  p21  p27  CDK2  Gata6  Brachyury  Map2  Cdx2  Sall4  GAPDH | Forward CTG AGG GCC AGG CAG GAG CAC GAG  Reverse CTG TAG GGA GGG CTT CGG GCA CTT  Forward GGT TAC CTC TTC CTC CCA CTC CAG  Reverse TCA CAT GTG CGA CAG GGG CAG  Forward ACG AGT GGC AGT TTC TTC TTG GGA  Reverse TAT GAC TCA CTT CCA GGG GGC ACT  Forward AGG GTC TGC TAC TGA GAT GCT CTG  Reverse CAA CCA CTG GTT TTT CTG CCA CCG  Forward TTA TGA AGC ACA CAG CCT GCA ATG T  Reverse ACG GAC AGC CAA CCA ACT AAC GG  Forward tca aac gtg aga gtg tct aac gg  Reverse ctc aga cac cag agt gc  Forward GGG CAG ATA CGA GTG GCA G  Reverse CCT GAG ACC CAA TTA AAG GCA C  Forward TGTGCCTCCCCTGGATGAAG  Reverse CATCCTGGAAGAAAGGGTGA  Forward ACC TTA TGG CGT AGA AAT GCT GAG GGT G  Reverse CTG AAT ACT TGA GGT CAC TGT TCT CGG G  Forward ATG CCA AAG AAA GAA ACG AC  Reverse AGA GGC TGT AGA ACA TGA TT  Forward CAT CGC CAG CCT CGG AAC AAA CAG  Reverse TGC GCA AAT GGA ACT GGA GGC AAC  Forward GGC GAA ACC TGT GCG AGT GGA TGC GGA A  Reverse GAT TGC TGT GCC GCC GCC GCT TCA GAC C  Forward AACATATGCGGGCGGGCCTTCA  Reverse CCAGGAGGCGGGGTCCACACTC  Forward GGT GCT GAG TAT GTC GTG GAG TCT A  Reverse CCT GCT TCA CCA CCT TCT TGA TGT C | Takahashi *et al.,* 2006  Takahashi *et al.,* 2006  Takahashi *et al.,* 2006  Takahashi *et al.,* 2006  Lu SJ *et al.,* 2002  yang W *et al.,* 2003  http: pga.mgh.harvar  d.edu/primerbank  Takahashi *et al.,* 2006  Takahashi *et al.,* 2006  Takahashi *et al.,* 2006  Takahashi *et al.,* 2006  [45]  **Yuan Y** *et al.,* 2001 |
